# Dublin Anti-Bullying Self-Efficacy Scales: Bifactor and Item Response Theory Models

**DOI:** 10.1177/08862605231155137

**Published:** 2023-03-03

**Authors:** Seffetullah Kuldas, Aikaterini Sargioti, James O’Higgins Norman

**Affiliations:** 1 Department of Media and Communication, University of Oslo, Oslo, Norway; 2 DCU Anti-Bullying Centre, Dublin City University, Dublin, Ireland

**Keywords:** bullying, victim, bystander, self-efficacy, bifactor, item response theory, measurement invariance

## Abstract

Dublin Anti-Bullying Self-Efficacy Scales aim to measure the effectiveness of school anti-bullying programs in promoting five steps that victims and bystanders take against online and offline bullying behaviors. These steps are anti-bullying self-efficacy beliefs to recognize bullying behaviors, comprehend emergency, take responsibility, know what to do, and intervene. However, when an anti-bullying program is very effective for the majority of participants who give high scores, a considerable number of participants who give low scores are very likely to be detected as outliers. This raises two measurement issues. First, high scores create highly negatively skewed data and lead to measuring a unidimensional rather than multidimensional construct. This could be one reason why recent research has been unclear about the extent to which the scales measure a unidimensional, multidimensional, or bifactor construct. Second, should outliers be removed or be considered as participants for whom the program was ineffective? If the scales had measurement invariance across the group of outliers and non-outliers or low and high self-efficacy, it could be concluded that the anti-bullying program was ineffective for some participants. The current research aims to address these issues by testing both measurement invariance as well as unidimensional and bifactor models of anti-bullying self-efficacy. Results of Pure Exploratory Bifactor (PEBI) Analyses and Item Response Theory (IRT) with Two-Parameter-Logistic (2PL) Models of data from a convenience sample of 14-year-old students in Ireland (*N* = 1,222) indicated sufficient psychometric properties of both unidimensional and multidimensional scales for victim offline, victim online, bystander offline, and bystander online. Further research can use these scales for measuring the bifactor model of anti-bullying self-efficacy as well as the cut-off score for distinguishing between low and high anti-bullying self-efficacy.

Online/offline aggressive behavior is defined as bullying when it (a) happens within a societal context, (b) causes physical, emotional, and/or indirect harm to the targeted person, and (c) depends on an imbalance of power that results from social/school/institutional norms or systems (UNESCO, 2020). A growing consensus on effective ways to prevent or intervene in bullying behaviors is that anti-bullying programs should focus more on promoting victims and bystanders’ anti-bullying self-efficacy beliefs (Sargioti et al., 2023). Anti-bullying self-efficacy refers to victims and bystanders’ confidence in their own ability, as well as the ability of teachers, parents, social and school environments (norms, systems, policies), to tackle online/offline bullying behaviours (Kuldas & Foody, 2022; Sargioti et al., 2023). For example, when bullied students have no confidence in teacher efficacy and attitude (e.g., believing that the school teacher will make the situation worse, not care, or take no action to prevent or intervene in bullying), they are unwilling to ask for help or disclose victimization (Mazzone et al., 2021).

However, the consensus has been falling short of theoretical and empirical evidence by lacking an anti-bullying self-efficacy framework and measurement scale (Sargioti et al., 2023). A recent literature review (Sargioti et al., 2023) found only one study (see Andreou et al., 2007) addressing the need for a framework and scale to assess the effectiveness of an anti-bullying program in terms of both victim and bystander’s self-efficacy, while other studies only focused on either victim’s self-efficacy (see Salimi et al., 2021) or bystander’s self-efficacy (see Knauf et al., 2018; Thornberg et al., 2017). Only recently, Sargioti and colleagues proposed both (a) *Anti-Bullying Self-Efficacy Scales* and (b) an *Anti-Bullying Self-Efficacy Theory*, which is a synthesis of the Participant Role Approach (Salmivalli et al., 1996) and Bystander Intervention Model (Latané & Darley, 1970). According to the proposed theory (Sargioti et al., 2023), anti-bullying self-efficacy is a mixture of individual and social capacity, process, and outcome of person-environment (student-teacher, child-parent, or peer-to-peer) transactions. For example, when victims have a caring and supportive teacher/parent/friend, they can demonstrate self-efficacy in tackling bullying behaviors (Kuldas & Foody, 2022). The theory hereby is not suggesting a trait-conception of self-efficacy as Bandura (1997) defined (the belief in individual ability to carry out a specific behavior in a successful way), because it lacks the account of social-ecological effects on the individual’s anti-bullying self-efficacy (Sargioti et al., 2023).

Unlike the trait-conception, the anti-bullying self-efficacy theory (Sargioti et al., 2023) provides a multidimensional conception and measurement scale for the identification of five steps that victims and bystanders take to intervene in online/offline bullying behaviors. These five steps are defined as dimensions of victim and bystander’s self-efficacy to: (a) *recognize* online/offline bullying behaviors, (b) *comprehend* the need for *emergency* intervention, (c) take *responsibility* for the intervention, (b) *know* what to do, and (e) *intervene* (Sargioti et al., 2023). The effectiveness of an anti-bullying program could be measured by the extent to which it has promoted anti-bullying self-efficacy beliefs pertaining to each step (Sargioti et al., 2023). However, when a school anti-bullying program is very effective, its participants give very high scores on a scale measuring the effectiveness (see Sargioti et al., 2023). In this case, the proposed scales can be misleading due to the three chief measurement issues below.

First, school anti-bullying programs are mostly focused on the rise/fall of bullying/victimization rates as a measure of their effectiveness, which is based on students’ self-reports as perpetrator, victim, or bystander after the implementation (Sargioti et al., 2023). A school anti-bullying program, which is usually focused on raising awareness, could be considered ineffective if students reported more incidents after the program (O’Moore & Minton, 2005). However, the higher rate can be a result of raised awareness about bullying behaviors rather than an actual increase in bullying incidents. Hence, the higher rate does not mean *ineffectiveness* but *effectiveness* of the anti-bullying program (Sargioti et al., 2023).

Second, the lowest rate also does not necessarily mean ineffectiveness of an anti-bullying program, because it could also be only for one dimension of anti-bullying self-efficacy beliefs, such as recognition or knowledge. This raises the issue of whether anti-bullying self-efficacy is measurable as a multidimensional or unidimensional construct. The extent to which the new scales allow for measuring each dimension alone and the general factor has remained unclear, mainly due to the lack of evidence for the dimensionality of anti-bullying self-efficacy construct. Recent results of four separate exploratory factor analyses (EFA) of the scales (Sargioti et al., 2023) displayed both: (a) the eigenvalue value of the first factor (e.g., 9.36 for the victim offline scale) was at least three times higher than the other factors in each scale (e.g., 2.84 for the second factor of the victim offline scale), and (b) at least three factors in each scale had considerable inter-factor correlations, ranging from .50 to .67. These results, as the ratio of the first to second eigenvalue >3.0 (Reise et al., 2010) and inter-factor correlations >.50 (Lorenzo-Seva & Ferrando, 2019a) or >.60 (García-Garzón et al., 2020), could be sufficient evidence for the co-existence of the general factor and sub-factors; therefore, the hypothesis of a bifactor model fit is appropriate to test (Reise et al., 2010). In addition, given that an accurate evaluation of school anti-bullying programs requires to account for students’ anti-bullying self-efficacy beliefs as both the general factor and sub-dimensions, a bifactor model of anti-bullying self-efficacy beliefs can be tested.

Third, the highest scores also stand for highly negatively skewed data. Statistical analysis of such non-normally distributed data is very likely to identify some participants as outliers who gave the lowest score on a scale for a single dimension (i.e., univariate outliers) or multiple dimensions (i.e., multivariate outliers) of a latent construct (Finch, 2012). In this case, does the lowest score mean that (a) the anti-bullying program was ineffective for some participants or (b) the measurement scale failed to distinguish between scores for low and high anti-bullying self-efficacy beliefs (i.e., lacking measurement invariance)? If the self-efficacy scale measured the same construct across outliers and non-outliers, it could be concluded that the anti-bullying program was ineffective for some participants. To test this hypothesis, a Two-Parameter-Logistic (2PL) Model of Item Response Theory (IRT) could be conducted to test item discrimination parameters and measurement invariance. The present paper presents statistical tests and results of unidimensional, bifactor, and IRT-2PL models.

## The Present Study

On the basis of the anti-bullying self-efficacy theory (Sargioti et al., 2023), the present research aims to address the abovementioned measurement issues by testing the unidimensional and bifactor models as well as the measurement invariance of item-responses to the four separate anti-bullying self-efficacy scales for victim offline, victim online, bystander offline, and bystander online. Although almost two decades ago, O’Moore and Minton (2005) drew attention to that school anti-bullying programs in Ireland need to focus on the enhancement and measurement of victim and bystander’s self-efficacy beliefs, research to address this need is still nascent. The effectiveness of school anti-bullying programs in Ireland is generally evaluated in terms of the prevalence rates of targets/perpetrators of bullying behaviors, raising awareness, anti-bullying policies, and a positive school climate (Foody et al., 2018). Such an evaluation leaves unclear the extent to which victim and bystander’s self-efficacy beliefs are effective in the prevention and/or intervention of online/offline bullying behaviors (Salimi et al., 2021). However, “there is a scarcity of published research on the measurement of both victim and bystander’s self-efficacy in bullying situations across countries, including Ireland” (Sargioti et al., 2023, p. 8). Research is needed for further validation of the new scales measuring the five steps of victim and bystander’s self-efficacy in tackling both offline and online bullying behavior (Sargioti et al., 2023).

Hence, the novel contribution of the present research ensues from both testing (a) the dimensionality of the anti-bullying self-efficacy construct and (b) measurement invariance, distinguishing between scores for low and high anti-bullying self-efficacy beliefs. The research hereby provides insights into the following questions:

- Is anti-bullying self-efficacy a bifactor construct?
- Do the anti-bullying scales distinguish between scores for low and high self-efficacy beliefs or measure the same construct across outliers and non-outliers?

## Methods

### Procedures

This research with a cross-sectional design is part of a wider anti-bullying program, implemented in post-primary schools in Ireland, the outline and results of which are not the main focus of the present study. Among all the invited post-primary schools in Ireland (*N* = 730), 355 expressed their interest in implementing it, but only 197 fully implemented it (October 2021–June 2022). Participating students were invited to complete an online survey about their self-efficacy in tackling online/offline bullying after the implementation of the program (Spring 2022). The survey link, along with instructions and consent forms, was sent to students and their parents via email. The ethics committee of the authors’ university granted ethical approval prior to the distribution of the survey and the program implementation.

### Participants and Settings

Participants were a convenience sample of 1,222 post-primary school students (14-year-old) in Ireland. However, due to outliers and random missing values, the final sample size varied by complete responses to each scale and statistical analyses. The final sample size differed for the single-unidimensional and bifactor modelling of the scale responses for victim offline (*N* = 1,041, Male = 44.1%, Female = 53.1%, Other = 2.8%), victim online (*N* = 1,028, Male = 43.6%, Female = 53.6%, Other = 2.8%), bystander offline (*N* = 1,061, Male = 43.6%, Female = 53.5%, Other = 2.8%), and bystander online (*N* = 1,022, Male = 43.3%, Female = 54.0%, Other = 2.6%) as well as the IRT-2PL model testing of the general and specific factor (*N* = 1,222, Male = 45.2%, Female = 50.9%, Other = 3.9%).

### Measures of Anti-bullying self-efficacy beliefs

The single-unidimensional and bifactor modelling of anti-bullying self-efficacy beliefs was based on item-responses (ranging from 5 – Very to 0 – Not at all) to the Dublin Anti-Bullying Self-Efficacy Scale for *victim offline* (20-item), *victim online* (20-item), *bystander offline* (20-item), and *bystander online* (20-item), developed by Sargioti et al. (2023). Each scale comprises five subscales: *recognition* (4-item), *emergency comprehension* (4-item), *responsibility* (4-item), *knowledge* (4-item), and *intervention* (4-item). Each subscale started with the statement “*The Anti-Bullying programme has increased my confidence in my ability. . .*” to recognize bullying behaviors, to comprehend emergency for intervention, to take responsibility, to know what to do, and to intervene (a) if I am bullied in person, (b) if I am bullied online, (c) if someone else is bullied in person, and (d) if someone else is bullied online. Regarding psychometric properties of the four scales, Sargioti et al. (2023) reported sufficient estimates of the content-face-construct validity and composite reliability.

### Statistical Assumptions and Data Analyses

The current research has conducted three main multivariate statistical analyses, testing unidimensional factor, bifactor, and IRT-2PL models. The unidimensional factor models were tested with four *separate* EFA, whereas bifactor models were tested with four *separate* Pure Exploratory Bifactor (PEBI) analyses using Robust Diagonally Weighted Least Squares (RDWLS), Promin rotation, closeness to unidimensionality test, and goodness-of-fit indices as implemented in the FACTOR program (Lorenzo-Seva & Ferrando, 2019a). To improve the overall accuracy of unidimensional EFA and PEBI results by correcting for bias and skewness in the distribution of bootstrap estimates (Zhang & Browne, 2006), a bias-corrected and accelerated bootstrap with 500 samples and 95% confidence intervals (CIs) was also computed. Following the Bifactor analyses, the IRT with a 2PL model (i.e., item difficulty and discrimination parameters) was conducted to test construct validity of item-responses to the four anti-bullying self-efficacy scales, to determine a cut-off score for low and high anti-bullying self-efficacy, and to test if outliers were statistically not representative of the research population.

An initial item analysis, check of missing data, detection of outliers, and normality test (Boxplot) were conducted using IBM SPSS (IBM Corporation, Statistical Package for Social Sciences, Version 27). The FACTOR program, Version 12.01 (Ferrando & Lorenzo-Seva, 2017) was used for the EFA and PEBI. For the IRT-2PL model testing, *Stata Statistical Software*—Release 17 (StataCorp, 2021) was used. A Microsoft excel-based tool (Dueber, 2017) and BifactorCalc online software (Ventura-León et al., 2021) were also used as calculators of bifactor dimensionality indices, especially for estimating Omega-Hierarchical (ω_H_) and Omega-Hierarchical-Subscale (ω_HS_) coefficients, and the Percent of Uncontaminated Correlations (PUC).

Before performing the statistical methods, their assumptions were tested. This was followed by assessments of criteria for factorability, factor analysis-rotation–extraction–retention, factor reliability, factor dimensionality, and robust goodness-of-fit statistics. The following subsections present statistical assumptions/indices and reasons for why the three multivariate statistical methods were chosen.

#### Outlier detection method: regression factor scores

A dataset is suitable for EFA when it satisfies statistical assumptions for handling missing data, outliers, normality, and multicollinearity (Tabachnick & Fidell, 2013). In particular, because outliers bias the sample mean score and can inflate an inter-factor correlation value (Brown, 2006), they should be removed prior to EFA when there is a theoretical reason (Field, 2013). However, given that outliers are peculiar to each study using EFA, a standardized score for outlier detection may facilitate the replicability of findings. One standardized method is to weight item scores according to their relationships to each factor and, thereby, create factor scores of each case/participant that can subsequently be included in further analysis (Watkins, 2021). This method is commonly applied by using *regression factor scores* that indicate the location of each case/participant’s relative standing on a latent common factor (DiStefano et al., 2009). Factor scores range approximately between −3.0 and +3.0, indicating standard deviation (*SD*) values below and above the mean (DiStefano et al., 2009). To use a specific range of regression factor scores could allow further research to test replicability of the same number of factors. To this aim, the present research used a regression factor score of ≥−2.0 (i.e., two *SD* below the mean), as it was the cut-off point inflating inter-factor correlations in the current dataset, and compared it with univariate outliers detected through *Boxplot* as well as with multivariate outliers detected through Mahalanobis’ (*D*^2^) distance (Field, 2013). Outliers that appeared on two tests were not included in EFA and PEBI but used as a criterion binary variable for testing measurement invariance to distinguish between the two fundamental issues as to whether the lowest score of some participants meant that the anti-bullying program was ineffective for them (or the anti-bullying scale could not differentiate between scores for low and high self-efficacy).

#### Factorability criteria

Factorability of item-responses to each scale was based on basic criteria for inter-item correlation (>.30 but <.90), strong pairwise correlation (for two and more items), and an adequate sample size (Tabachnick & Fidell, 2013). A Measure of Sampling Adequacy (MSA) was used first, which suggests removing an item <0.50 not measuring the same domain as the remaining items in the pool (Lorenzo-Seva & Ferrando, 2021). Next, the strength and adequacy of pairwise correlations were estimated through Bartlett’s (1954) test of sphericity (*p* < .05) and Kaiser–Meyer–Olkin (KMO) index > .50 (Tabachnick & Fidell, 2013). Last, the sample size was considered adequate if one common factor had four or more items with loading values of 0.60 (Jung et al., 2020) or each sub-factor explained a substantial proportion of variance with ω_HS_ ≥ .30 (Smits et al., 2014).

#### Bifactor analysis method

PEBI was used for the current dataset. Unlike traditional bifactor models, PEBI allows sub-factors to be correlated, to set a specific bifactor model a priori (Lorenzo-Seva & Ferrando, 2019a), to estimate *construct-relevant* multidimensionality in a set of ordered-categorical item-responses (Reise, 2012), and to produce unidimensionality estimates for IRT (Reise et al., 2013).

#### Factor rotation method

Given that the self-efficacy dimensions are theoretically correlated, the oblique rotation method used was Robust Promin (RB). RB produces pattern loading matrices that better approximate a simple unidimensional or bifactor structure (Lorenzo-Seva & Ferrando, 2019b).

#### Factor extraction method

RDWLS was used for the current dataset; it uses a polychoric correlation matrix, provides more accurate parameter estimates, and yields a robust model fit when a dataset lacks univariate and multivariate normal distributions (Mîndrilã, 2010). For three and more factors, RDWLS estimates factor loadings, standard errors, and factor correlations most precisely, closest to the true model (Mîndrilã, 2010).

#### Factor retention criteria

For the EFA and PEBI, six basic criteria were initially considered to retain a factor having a model fit index, three or more items, adequate item-loading, convergent validity, internal consistency, and construct reliability. First, the number of factors was estimated with a Schwarz Bayesian Information Criterion (BIC) dimensionality test, a theoretically suitable and more robust simplistic model (Neath & Cavanaugh, 2012) in the FACTOR program (Gibson et al., 2020). The smaller the BIC value, the more probable the statistical model is an accurate fit for the given data (Neath & Cavanaugh, 2012). Second, to reflect or identify one factor, the minimum number of items is three (Brown, 2006) or two in oblique rotation (Abad et al., 2017). However, four items are recommended to reflect factor content (Robertson, 2019). Third, a minimum value of 0.32 for item-loading is acceptable (Tabachnick & Fidell, 2013). A cross-loading of one item on a factor should have a value of 0.20 greater than all of its loadings on other factors (Cabrera-Nguyen, 2010). An item with loading <0.32 and cross-loading <0.20 should be removed. Similar item-loading values are applicable to a bifactor analysis. A bifactor model is considered *unsuitable* where (a) no item of a sub-factor shows loading value >0.20 on the general factor, (b) the general factor mainly shows either *lower* or *higher* loading values than the sub-factor loadings, (c) there is no theoretical justification, and (d) goodness-of-fit indices show a poor model fit (Lorenzo-Seva & Ferrando, 2019a). If an item-loading on a sub-factor is high (>.80), that item should have a smaller loading value on the general factor, or the reverse (Robertson, 2019). An item should not have equal or very high loadings on both general and sub-factor (Robertson, 2019). Zero cross-loadings indicate a very accurate estimation of loadings on both general factor and sub-factors (Reise, 2012). Higher loadings (of most items) on the general factor than the sub-factor suggest using the general factor score is more appropriate (Robertson, 2019). Fourth, as a common measure to estimate convergent validity is the Average Variance Extracted (AVE > .50), the sum of the squared loadings divided by the number of indicators (Hair et al., 2014). The AVE from each unidimensional factor scale was estimated to assess convergent validity as one criterion for construct validity. Fifth and sixth were the following internal consistency and construct replicability criteria.

#### Factor reliability estimation methods

An initial estimation of internal consistency of unidimensional factors was based on the Omega Index (≥.80), the factorial loads rather than the number of items (McDonald, 1999). For bifactors, estimations of the internal consistency and relative strength of the general factor and sub-factors were respectively based on Omega-Hierarchical (ω_H_) and Omega-Hierarchical-Subscale (ω_HS_) coefficients (Reise et al., 2010; Rodriguez et al., 2016b). The ω_H_ represents the proportion of variance in the total score that can be attributed to the general factor *after accounting for all sub-dimensions* (Reise et al., 2013). A value of ω_H_ ≥ .50 is acceptable, but closer to .75 is preferred (Reise et al., 2013). Higher values ω_H_ ≥ .80 indicates that the latent construct can be considered essentially unidimensional (Reise et al., 2010; Ventura-León et al., 2021). As to the ω_HS_, it stands for the proportion of systematic variance in a subscale data that can be uniquely attributed to a sub-dimension *after accounting for the general factor* (Rodriguez et al., 2016b). A cut-off score ω_HS_ ≥ .30 is substantial, ≤.29 to −.20 is moderate, and ≤.19 is low (Smits et al., 2014) proportion of unique variance explained by a subscale (i.e., unique variance not explained by the general factor). Another estimation of internal consistency is the construct replicability of the general factor and sub-factors, measuring the extent to which a latent construct is reproducible (replicable) from its own indicators (Hancock & Mueller, 2001). Hancock’s and Mueller’s construct reliability index (*H* ≥ .70) was used; it indicates a well-defined latent construct, which is more likely to be stable or replicable across studies.

#### Factor dimensionality assessments: single factor versus bifactor

The unidimensionality assessments of both single factor and bifactor models were based on seven criteria: Unidimensional Congruence (UniCo), Item Unidimensional Congruence (I-Unico), Explained Common Variance (ECV), Item Explained Common Variance (I-ECV), Mean of Item Residual Absolute Loadings (MIREAL), Item Residual Absolute Loadings (I-REAL), and PUC. Values of UniCo and I-Unico > 0.95, ECV and I-ECV > 0.85, MIREAL and I-REAL < 0.300 suggest that the latent construct can be treated as essentially unidimensional (Ferrando & Lorenzo-Seva, 2018, 2019). A value of ECV < 0.70 and higher ω_HS_ indicates a multidimensional model (Rodriguez et al., 2016a).

If unidimensionality is unclear (i.e., if exploratory common factor analysis suggests the presence of sub-dimensions), a comparison of PUC, ECV, and ω_H_ values is needed (Reise et al., 2013). Although there is no consensus over a cut-off score for this comparison, there are two suggestions to treat a latent construct as unidimensional (Ventura-León et al., 2021). When PUC is <.70, ECV *should be* > .70 and ω_H_ > .80 (Rodriguez et al., 2016a; 2016b); or when PUC is <.80, ECV *should be* > .60 and ω_H_ > .70 (Reise et al., 2013). When PUC is >.80, a unidimensional model is possible to consider, even if a bifactor model better fits the data (Ventura-León et al., 2021). PUC > .80 but not >.90 indicates that the size of unidimensionality is high but not to a severe degree to rule out multidimensionality, thereby qualifying a latent construct as a bifactor (Reise et al., 2013; Ventura-León et al., 2021). When PUC is very high (>.90), “the parameter estimates in the unidimensional model are the same as the general factor in the bifactor model” (Reise, 2012, p. 688).

As to identify which items contribute more to *the general factor* than a sub-factor, the criterion reference was I-ECV > 0.85 that indicates an influence of the general factor on the item variance (Stucky & Edelen, 2014). I-ECV near 1 indicates that an item solely reflects the general factor (Ferrando & Lorenzo-Seva, 2018).

#### Robust goodness-of-fit indices

To estimate the extent to which either the single-unidimensional or bifactor model approximates reality, goodness-of-fit indices were: Robust Mean and Variance-Adjusted Chi Square with Degree of Freedom, χ^2^/*df* = < 3 (Mîndrilã, 2010), Adjusted Goodness-of-Fit Index (AGFI) > .90, Comparative Fit Index (CFI > .95), Non-Normed Fit Index (NNFI, also known as TLI = Tucker-Lewis coefficient) > .95, Root Mean Square of Residuals (RMSR) < 0.08 lower than Kelley’s criterion value (Ferrando & Lorenzo-Seva, 2017), and Root Mean Square Error of Approximation (RMSEA) < 0.08 (Mîndrilã, 2010). In brief, a unidimensional or bifactor model fit was acceptable only with χ^2^*/df* ≤ 3.0, RMSEA ≤ 0.08, RMSR ≤ 0.08, and AGFI, CFI, NNFI/TLI ≥ 0.95 (Lorenzo-Seva & Ferrando, 2019a).

#### IRT-2PL model

In order to perform the IRT-2PL model, all the items were coded as binary, based on the item discrimination test of each point within the 6-point scale, until the common cut-off score (between 0 and 5, discriminating between low and high anti-bullying self-efficacy as the general and specific factor), was found and displayed by the *Test Characteristic Curve* (TCC). TCC illustrates individuals’ latent characteristics based on their true scores on a measurement scale (Baker, 2001), thereby displaying the discriminating ability (cut-off points) for determining anti-bullying self-efficacy levels. The scale points of 0, 1, and 2 were re-coded as 0 for low self-efficacy, whereas 3, 4, and 5 were re-coded as 1 for high self-efficacy. To test measurement invariance across sample characteristics, a binary variable for detected outliers (0) and non-outliers (1) as well as for male (0) and female (1) samples was created.

Four assumptions for the IRT-2PL analyses of both general and specific factors were met. Assumption 1, unidimensionality was based on statistical criteria outlined in the FACTOR statistical program (Ferrando & Lorenzo-Seva, 2018). Assumption 2, local independence was based on coefficient Loevinger’s *H* > 0.30, using the *mokken* package in R (van der Ark, 2010). Assumption 3, monotonicity was displayed on a graph with an *S* shape curve (Yang & Kao, 2014). Assumption 4, measurement invariance across groups of detected outliers and non-outliers as well as male and female samples was estimated through Lord’s chi-squared method for Differential Item Functioning (DIF, Lord, 1980), as implemented in Stata Statistical Software—Release 17 (StataCorp, 2021).

## Statistical Results

Statistical assumptions for EFA of item-responses to the self-efficacy scales for victim offline, victim online, bystander offline, and bystander online were met satisfactorily after removing missing data (4% cases with a missing value for any variable) via *listwise* deletion (Field, 2013) and multivariate/univariate outliers, which inflated inter-factor correlations and had lower scores (*Mean* < 2.1). The cases of outliers on the scale for victim offline (*n* = 181, 14.8%), victim online (*n* = 194, 15.9%), bystander offline (*n* = 161, 13.2%), and bystander online (*n* = 200, 14.4%) were not included in the EFA of each dataset (*N* = 1,222) respectively. The sample size was adequate, as the 20-item of each general factor had a minimum loading value of 0.60 (Jung et al., 2020) and the 4-item of each sub-factor had ω_HS_ ≥ .30 (Smits et al., 2014).

The inter-item correlation indices of the four scales displayed linearity among pairs of items (*r* > .30 but not >.90), indicating some collinearity but not to the extent of multicollinearity (Tabachnick & Fidell, 2013). Although values for multivariate skewness and kurtosis were <±3 (Mîndrilã, 2010), analyses of the Mardia’s multivariate normality indicated a non-significant skewness but significant kurtosis of the item-responses to the scale for victim offline (Skewness: χ^2^ = 2,413, *df* = 1,540, *p* > .99; Kurtosis: χ^2^ = 370, *p* < .05), victim online (Skewness: χ^2^ = 2,119, *df* = 1,540, *p* > .99; Kurtosis: χ^2^ = 334, *p* < .05), bystander offline (Skewness: χ^2^ = 1,780, *df* = 1,540, *p* > .99; Kurtosis: χ^2^ = 276, *p* < .05), and bystander online (Skewness: χ^2^ = 2,118, *df* = 1,540, *p* > .99; Kurtosis: χ^2^ = 339, *p* < .05). These non-normal distributions required using polychoric correlation matrices, so that the model chosen could fit the data (Lorenzo-Seva & Ferrando, 2019a). Every item on each scale had an MSA value greater than 0.92, suggesting no item removal (Lorenzo-Seva & Ferrando, 2021). The KMO measure along with Bartlett’s test of sphericity verified the sampling adequacy for EFA of item-responses to the scale for victim offline (KMO = .94; Bartlett’s statistic: 11887.1, *df* = 190, *p* < .001), victim online (KMO = .95; Bartlett’s statistic: 11737.4, *df* = 190, *p* < .001), bystander offline (KMO = .95; Bartlett’s statistic: 12117.4, *df* = 190, *p* < .001), and bystander online (KMO = 95; Bartlett’s statistic: 11668.3, *df* = 190, *p* < .001).

### Results for Exploratory Unidimensional Factor Models

Table 1 displays statistical values of item-loading, dimensionality, reliability, construct replicability, and goodness-of-fit indices for all the four unidimensional and bifactor models.

**Table 1.** Results of Exploratory Factor Analyses of the Four Unidimensional Models and PEBI Analyses of the Four Anti-Bullying Self-Efficacy Scales.

Factor	Item	Unidimensional Model				Bifactor Model								Unidimensional Model				Bifactor Model							
		Single Factor λ	I-ECV	I-Unico	I-REAL	General Factor λ	I-ECV	I-Unico	F1 λ	F2 λ	F3 λ	F4 λ	F5 λ	Single Factor λ	I-ECV	I-Unico	I-REAL	General Factor λ	I-ECV	I-Unico	F1 λ	F2 λ	F3 λ	F4 λ	F5 λ
		if I am bullied in person (Victim Offline, *N* = 1,041)												if I am bullied online (Victim Online, *N* = 1,028)											
Recognition	1. To be aware	.655	0.644	0.875	0.480	.607	0.519	0.699	.613					.696	0.598	0.830	0.547	.593	0.464	0.621				.665	
	2. To realize	.677	0.599	0.831	0.536	.610	0.469	0.639	.669					.695	0.574	0.803	0.570	.625	0.503	0.664				.662	
	3. To notice	.702	0.642	0.874	0.513	.672	0.575	0.775	.607					.720	0.615	0.848	0.535	.650	0.527	0.709				.648	
	4. To recognize bullying behaviors	.679	0.596	0.828	0.543	.600	0.465	0.662	.638					.713	0.571	0.799	0.585	.655	0.536	0.720				.641	
Emergency Comprehension	5. To see the need to ask for help	.741	0.975	0.999	0.120	.684	0.757	0.956			.379			.723	0.990	0.999	0.073	.648	0.671	0.905	.444				
	6. To see the need to tell someone	.774	0.995	0.999	0.055	.694	0.605	0.815			.586			.760	0.999	0.999	0.004	.710	0.658	0.871	.533				
	7. To see the need for urgent help	.758	0.967	0.999	0.136	.729	0.686	0.892			.518			.754	0.999	0.999	0.002	.666	0.560	0.771	.605				
	8. To see the need to take action	.749	0.981	0.999	0.103	.613	0.480	0.684			.633			.739	0.998	0.999	0.037	.698	0.619	0.833	.563				
Responsibility	9. To take responsibility for speaking out	.738	0.992	0.999	0.064	.723	0.698	0.911				.485		.756	0.976	0.999	0.119	.621	0.496	0.793			.542		
	10. To take responsibility for reporting	.743	0.989	0.999	0.079	.703	0.623	0.844				.559		.754	0.945	0.998	0.183	.699	0.636	0.868			.529		
	11. To take responsibility for telling someone	.745	0.972	0.999	0.128	.633	0.523	0.753				.590		.771	0.940	0.998	0.192	.736	0.666	0.884			.535		
	12. To take responsibility for taking action	.735	0.975	0.999	0.117	.639	0.532	0.753				.597		.748	0.956	0.999	0.158	.701	0.622	0.843			.560		
Knowledge	13. To know where to report	.703	0.878	0.991	0.264	.720	0.700	0.913					.481	.702	0.971	0.999	0.123	.580	0.471	0.764		.532			
	14. To know what to do	.698	0.883	0.991	0.255	.629	0.550	0.770					.573	.701	0.958	0.999	0.150	.642	0.576	0.808		.548			
	15. To know whom to ask for help	.702	0.851	0.985	0.296	.666	0.563	0.775					.601	.709	0.928	0.997	0.199	.683	0.604	0.817		.574			
	16. To know how to report	.673	0.845	0.984	0.291	.545	0.399	0.579					.647	.687	0.929	0.997	0.193	.701	0.703	0.910		.472			
Intervention	17. To tell someone	.712	0.930	0.997	0.197	.702	0.709	0.909		.475				.727	0.936	0.998	0.190	.585	0.448	0.688					.600
	18. To ask for help	.739	0.922	0.996	0.217	.691	0.691	0.925		.443				.747	0.956	0.999	0.162	.660	0.595	0.849					.520
	19. To report	.739	0.912	0.995	0.228	.658	0.540	0.755		.613				.732	0.879	0.991	0.274	.713	0.657	0.888					.513
	20. Where to report	.748	0.935	0.998	0.198	.638	0.509	0.737		.611				.725	0.918	0.996	0.218	.739	0.708	0.927					.470
	Unidimensionality Indices (95% CI)																								
	UniCo	0.97 [0.95, 0.98]				0.79 [0.78, 0.80]								0.96 [0.95, 0.98]				0.81 [0.80, 0.82]							
	ECV	0.86 [0.85, 0.88]				0.58 [0.01, 0.59]								0.86 [0.85, 0.88]				0.59 [0.56, 0.60]							
	MIREAL	0.24 [0.21, 0.27]												0.23 [0.20, 0.25]											
	PUC					.84												.84							
	ω_T_	.91												.91											
	ω_H_					.85												.86							
	ω_HS_								.48	.36	.35	.38	.41								.35	.36	.36	.49	.35
	*H*	.99				.90			.76	.71	.70	.72	.77	.99				.91			.70	.73	.74	.74	.71
	AVE	0.52												0.53											
	Goodness-of-Fit Indices (95% CI)																								
	χ^2^/*df*	206/170 = 1.21, *p* < .001				114/85 = 1.34, *p* < .05								274/170 = 1.61, *p* < .001					102/85 = 1.20, *p* < .05						
	AGFI	.99 [0.99, 0.99]				.99 [0.99, 1.00]								.99 [0.99, 0.99]					.99 [0.99, 1.00]						
	CFI	.97 [0.96, 0.97]				.99 [0.99, 1.00]								.96 [0.95, 0.97]					.99 [0.99, 1.00]						
	TLI/NNFI	.96 [0.95, 0.97]				.99 [0.99, 1.00]								.95 [0.94, 0.96]					.99 [0.99, 1.00]						
	RMSR	0.11 [0.10, 0.12]				0.02 [0.02, 0.02]								0.11 [0.09, 0.12]					0.02 [0.02, 0.02]						
	RMSEA	0.10 [0.09, 0.11]				0.02 [0.00, 0.02]								0.12 [0.11, 0.14]					0.01 [0.11, 0.13]						
Factor	Item	Unidimensional Model				Bifactor Model								Unidimensional Model				Bifactor Model							
		Single Factor λ	I-ECV	I-Unico	I-REAL	General Factor λ	I-ECV	I-Unico	F1 λ	F2 λ	F3 λ	F4 λ	F5 λ	Single Factor λ	I-ECV	I-Unico	I-REAL	General Factor λ	I-ECV	I-Unico	F1 λ	F2 λ	F3 λ	F4 λ	F5 λ
		If someone else is bullied in person (Bystander Offline, *N* = 1,061)												If someone else is bullied online (Bystander Online, *N* = 1,022)											
Recognition	1. To be aware	.712	0.638	0.870	0.514	.593	0.434	0.604					.681	.736	0.719	0.931	0.440	.657	0.527	0.716		.648			
	2. To realize	.698	0.604	0.837	0.533	.634	0.512	0.682					.656	.719	0.696	0.916	0.461	.644	0.522	0.706		.650			
	3. To notice	.785	0.672	0.899	0.527	.730	0.610	0.835					.592	.729	0.697	0.917	0.461	.652	0.526	0.725		.636			
	4. To recognize bullying behaviors	.782	0.665	0.893	0.527	.725	0.608	0.829					.595	.710	0.688	0.910	0.461	.640	0.518	0.701		.647			
Emergency Comprehension	5. To see the need to ask for help	.821	0.987	0.999	0.091	.703	0.621	0.882			.513			.724	0.928	0.997	0.203	.650	0.629	0.875				.483	
	6. To see the need to tell someone	.811	0.991	0.999	0.078	.735	0.668	0.876			.545			.778	0.979	0.999	0.112	.723	0.646	0.865				.550	
	7. To see the need for urgent help	.783	0.991	0.999	0.074	.720	0.623	0.819			.603			.759	0.938	0.998	0.192	.674	0.544	0.759				.624	
	8. To see the need to take action	.816	0.991	0.999	0.078	.760	0.720	0.913			.507			.759	0.954	0.999	0.165	.712	0.638	0.856				.553	
Responsibility	9. To take responsibility for speaking out	.814	0.981	0.999	0.114	.727	0.632	0.879				.534		.761	0.992	0.999	0.066	.640	0.526	0.764	.588				
	10. To take responsibility for reporting	.758	0.987	0.999	0.091	.727	0.660	0.876				.539		.780	0.997	0.999	0.043	.713	0.626	0.852	.559				
	11. To take responsibility for telling someone	.813	0.990	0.999	0.086	.757	0.648	0.872				.566		.790	0.999	0.999	0.013	.685	0.585	0.829	.562				
	12. To take responsibility for taking action	.800	0.992	0.999	0.074	.734	0.696	0.923				.472		.773	0.999	0.999	0.015	.761	0.712	0.924	.489				
Knowledge	13. To know where to report	.704	0.891	0.993	0.245	.562	0.414	0.615		.636				.713	0.768	0.957	0.388	.655	0.545	0.760					.605
	14. To know what to do	.795	0.918	0.996	0.243	.721	0.652	0.883		.526				.706	0.805	0.972	0.346	.658	0.567	0.777					.592
	15. To know whom to ask for help	.709	0.902	0.994	0.237	.680	0.638	0.832		.555				.695	0.783	0.964	0.363	.605	0.454	0.643					.660
	16. To know how to report	.692	0.880	0.991	0.253	.727	0.748	0.934		.443				.652	0.803	0.971	0.328	.648	0.647	0.859					.500
Intervention	17. To tell someone	.767	0.921	0.996	0.227	.615	0.484	0.765	.563					.727	0.914	0.996	0.228	.602	0.485	0.736			.577		
	18. To ask for help	.694	0.811	0.974	0.336	.650	0.664	0.923	.417					.726	0.880	0.991	0.272	.623	0.528	0.797			.542		
	19. To report	.791	0.936	0.998	0.206	.760	0.713	0.911	.511					.739	0.900	0.994	0.246	.733	0.664	0.881			.537		
	20. Where to report	.833	0.945	0.998	0.199	.790	0.734	0.931	.495					.723	0.939	0.998	0.185	.748	0.738	0.934			.462		
	Unidimensionality Indices (95% CI)																								
	UniCo	0.97 [0.96, 0.98]				0.84 [0.83, 0.85]								0.98 [0.97, 0.99]				0.80 [0.79, 0.84]							
	ECV	0.88 [0.86, 0.89]				0.64 [0.14, 0.64]								0.86 [0.85, 0.88]				0.58 [0.04, 0.59]							
	MIREAL	0.24 [0.21, 0.26]												0.25 [0.21, 0.27]											
	PUC					.84												.84							
	ω_T_	.96												.92											
	ω_H_					.88												.86							
	ω_HS_								.31	.36	.34	.32	.45								.36	.48	.35	.37	.42
	*H*	.97				.90			.72	.71	.70	.75	.75	.99				.91			.72	.79	.70	.73	.75
	AVE	0.59												0.54											
	Goodness-of-Fit Indices (95% CI)																								
	χ^2^/*df*	240/170 = 1.41, *p* < .001				172/85 = 2.02, *p* < .001								244/170 = 1.43, *p* < .001						242/85 = 2.84, *p* < .001					
	AGFI	.99 [0.99, 0.99]				.99 [0.99, 1.00]								.99 [0.99, 0.99]						.99 [0.99, 1.00]					
	CFI	.98 [0.97, 0.98]				.99 [0.99, 1.00]								.96 [0.95, 0.97]						.99 [0.99, 1.00]					
	TLI/NNFI	.97 [0.97, 0.98]				.99 [0.99, 1.00]								.95 [0.94, 0.96]						.99 [0.99, 1.00]					
	RMSR	0.10 [0.09, 0.11]				0.02 [0.02, 0.02]								0.11 [0.10, 0.12]						0.02 [0.02, 0.02]					
	RMSEA	0.11 [0.09, 0.11]				0.03 [0.00, 0.04]								0.13 [0.11, 0.14]						0.04 [0.02, 0.05]					

*Note.* The extraction method was Robust Diagonally Weighted Least Squares with Robust Promin, based on Bias-Corrected and Accelerated Bootstrap (500 samples) and 95% Confidence Intervals (CI). Single and general factor loadings (λ) were above .60. Reliability of unidimensional factors was estimated via McDonald’s Omega-Total (ω), whereas the general factor and sub-factors were estimated via Omega-Hierarchical (ω_H_) and Omega Hierarchical-Subscale (ω_HS_) respectively. Construct replicability test was based on Hancock’s and Mueller’s construct reliability index (*H*). Convergent validity of unidimensional factors was estimated via AVE (AVE = the sum of the squared loadings divided by the number of indicators). UniCo = Unidimensionality assessments were based on Unidimensional Congruence; I-Unico = Item Unidimensional Congruence, ECV = Explained Common Variance; I-ECV = Item Explained Common Variance; MIREAL = Mean of Item Residual Absolute Loadings; I-REAL = Item Residual Absolute Loadings; PUC = Percent of Uncontaminated Correlations. Estimations of goodness-of-fit were based on Robust Mean and Variance-Adjusted Chi Square with Degree of Freedom (χ^2^/df), AGFI = Adjusted Goodness-of-Fit Index; CFI = Comparative Fit Index; Non-Normed Fit Index (NNFI/TLI = Tucker-Lewis coefficient), RMSR = Root Mean Square of Residuals; RMSEA = Root Mean Square Error of Approximation.

#### Dimensionality

Assessments of the closeness to unidimensionality of the single factor model with 95% CI yielded sufficient values of UniCo and I-Unico > 0.95, ECV and I-ECV > 0.85, MIREAL and I-REAL < 0.300 for all the scales and respective items, except Item 1, 2, 3, and 4 (Ferrando & Lorenzo-Seva, 2018, 2019). Hence, anti-bullying self-efficacy was essentially a unidimensional construct on 16 items (from Item 5 to 20) of the scale for:

- *victim offline* (UniCo = 0.97 [95% CI: 0.95, 0.98], I-Unico > 0.95, ECV = 0.86 [0.85, 0.88], I-ECV > 0.85, MIREAL = 0.24 [0.21, 0.27], and I-REAL < 0.30);
- *victim online* (UniCo = 0.96 [95% CI: 0.95, 0.98], I-Unico > 0.99, ECV = 0.86 [0.85, 0.88], I-ECV > 0.87, MIREAL = 0.23 [0.20, 0.25], and I-REAL < 0.30);
- *bystander offline* (UniCo = 0.97 [95% CI: 0.96, 0.98], I-Unico > 0.97, ECV = 0.88 [0.86, 0.89], I-ECV > 0.85 (except Item 18), MIREAL = 0.24 [0.21, 0.26], and I-REAL < 0.30 (except Item 18); and
- *bystander online* (UniCo = 0.98 [95% CI: 0.97, 0.99], I-Unico > 0.95, ECV = 0.86 [0.85, 0.88], I-ECV > 0.85 (except Item 13, 14, 15, and 16), MIREAL = 0.25 [0.21, 0.27], and I-REAL < 0.30, except Item 13, 14, 15, and 16).

#### Reliability and validity

Values for the total omega and AVE from the unidimensional item-responses to the scale for victim offline (ω = .91; AVE = 0.52), victim online (ω = .91; AVE = 0.53), bystander offline (ω = .96; AVE = 0.59), and bystander online (ω = .92; AVE = 0.54) indicated high internal consistency and convergent validity (Hair et al., 2014). The *H* values ≥ 0.70 for construct replicability (Hancock & Mueller, 2001) also indicated that the 20 items for victim offline (*H* = 0.99 with 95% CI: [0.99, 1.00]), victim online (*H* = .99, [0.99, 1.00]), bystander offline (*H* = 0.99, [0.96, 0.97]), and bystander online (*H* = 0.99, [1.00, 1.01]) represented a unidimensional anti-bullying self-efficacy construct, which could be stable across studies.

#### Goodness-of-fit

Unlike the unidimensional factor results, robust goodness-of-fit indices with 95% CI suggested not considering the unidimensional model as good with the value of RMSR greater than the Kelley’s criterion and RMSEA > 0.08, although the other indices were good with χ^2^/*df* ≤ 3 and AGFI, CFI, NNFI/TLI ≥ 0.95 (Ferrando & Lorenzo-Seva, 2019a) for the item-responses to the scale for:

- *victim offline* (RMSR = 0.11 > Kelley’s value = 0.03 [95% CI: 0.10, 0.12], RMSEA = 0.10 [0.09, 0.11], χ^2^*/df* = 206/170 = 1.21 [<3, *p* < .001], AGFI = .99 [0.99, 0.99], CFI = .97 [0.96, 0.97], TLI/NNFI = 0.96 [0.95, 0.97]);
- *victim online* (RMSR = 0.11 > Kelley’s value = 0.03 [95% CI: 0.09, 0.12], RMSEA = 0.12 [0.11, 0.13], χ^2^/*df* = 274/170 = 1.61 [<3, *p* < .001], AGFI = .99 [0.99, 0.99], CFI = 0.96 [0.95, 0.97], TLI/NNFI = 0.95 [0.96, 0.96]);
- *bystander offline* (RMSR = 0.10 > Kelley’s value = 0.03 [95% CI: 0.09, 0.11], RMSEA = 0.11 [0.09, 0.11], χ^2^/*df* = 240/170 = 1.41 [<3, *p* < .001], AGFI = .99 [0.99, 0.99], CFI = .98 [0.97, 0.98], TLI/NNFI = 0.97 [0.97, 0.98]); and
- *bystander online* (RMSR = 0.11 > Kelley’s value = 0.03 [95% CI: 0.10, 0.12], RMSEA = 0.13 [0.11, 0.14], χ^2^/*df* = 318/170 = 1.71 [<3, *p* < .001, AGFI = 0.99 [0.99, 0.99], CFI = 0.96 [0.95, 0.97], TLI/NNFI = 0.95 [0.94, 0.96]).

Given that Item 1, 2, 3, 4 appeared to be not unidimensional and the BIC dimensionality test also resulted in a five-factor multidimensional solution for the item-responses to the scale for victim offline (BIC = 967.28), victim online (BIC = 987.69), bystander offline (BIC = 1045.91), and bystander online (BIC = 1091.24), a bifactor model could be tested.

### Results for Exploratory Bifactor Models

#### Loadings

Preliminary evidence for the bifactor model was observed by the item-loading values ranging from 0.38 to 0.67 on all the sub-factors and from 0.60 to 0.75 on all the general factors (Ferrando & Lorenzo-Seva, 2018). Factor loadings were significantly larger on the general factor than the sub-factors (see Table 1). Only the scale Item 1, 2, 3, 8, and 16 for victim offline; Item 1, 2, and 17 for victim online; Item 1, 2, and 13 for bystander offline; and Item 2, 4, and 15 for bystander online loaded on the respective sub-factors more than the general factor.

#### Dimensionality

The UniCo to I-Unico ≤ 0.95 and ECV to I-ECV ≤ 0.85 with 95% CI (Ferrando & Lorenzo-Seva, 2018, 2019) suggested that the latent construct could *not* be treated as essentially unidimensional. The highest value of ECV ≤ 0.64 by the general factor and the I-ECV ≤ 0.76 suggested that all the four scale data for anti-bullying self-efficacy were sufficiently multidimensional to warrant a bifactor model. Items having an I-ECV < 0.85 were considered measuring the respective sub-factor more than the general factor (Stucky & Edelen, 2014).

However, all the four PUC values were = .84 > .80 (Reise et al., 2013), 84% of the inter-item total correlations were *uncontaminated* by the multidimensionality. In other words, 84% of the common variance was explained by the general factor alone, whereas the rest 16% was explained by the multidimensionality (Ventura-León et al., 2021). The high percentage of unidimensionality and low percentage of multidimensionality could still be considered sufficient to qualify the anti-bullying self-efficacy construct as a bifactor on the scale for:

- *victim offline* (UniCo = 0.79 [95% CI: 0.78, 0.80], I-Unico < 0.93, ECV = 0.58 [0.01, 0.59], I-ECV < 0.76, and PUC = .84);
- *victim online* (UniCo = 0.81 [95% CI: 0.80, 0.82], I-Unico < 0.93, ECV = 0.58 [0.56, 0.60], I-ECV < 0.71, and PUC = .84);
- *bystander offline* (UniCo = 0.79 [95% CI: 0.78, 0.80], I-Unico < 0.93, ECV = 0.64 [0.14, 0.64], I-ECV < 0.75, and PUC = .84); and
- *bystander online* (UniCo = 0.80 [95% CI: 0.79, 0.84], I-Unico < 0.93, ECV = 0.58 [0.04, 0.59], I-ECV < 0.74, and PUC = .84).

#### Reliability

Estimates of ω_H_ ≥ .80 indicated the general factor was the main source of variance in each scale (Reise et al., 2013). However, the values of ω_HS_ ≥ .30 (Smits et al., 2014) and *H* index ≥ .70 (Hancock & Mueller, 2001) indicated that anti-bullying self-efficacy could be treated as a sufficiently defined bifactor construct that would be replicable for:

- *victim offline* as the general factor (ω_H_ = .85; *H* = .85) and specific dimension: recognition (ω_HS_ = .48; *H* = .76), emergency comprehension (ω_HS_ = .35; *H* = .70), responsibility (ω_HS_ = .38; *H* = .72), knowledge (ω_HS_ = .41; *H* = .75), and intervention (ω_HS_ = .36; *H* = .71);
- *victim online* as the general factor (ω_H_ = .86; *H* = .86) and specific dimension: recognition (ω_HS_ = .49; *H* = .74), emergency comprehension (ω_HS_ = .35; *H* = .70), responsibility (ω_HS_ = .36; *H* = .74), knowledge (ω_HS_ = .36; *H* = .73), and intervention (ω_HS_ = .35; *H* = .71);
- *bystander offline* as the general factor (ω_H_ = .88; *H* = .88) and specific dimension: recognition (ω_HS_ = .45; *H* = .77), emergency comprehension (ω_HS_ = .34; *H* = .70), responsibility (ω_HS_ = .32; *H* = .75), knowledge (ω_HS_ = .36; *H* = .71), and intervention (ω_HS_ = .31; *H* = .72); and
- *bystander online* as the general factor (ω_H_ = .86; *H* = .86) and specific dimension: recognition (ω_HS_ = .48; *H* = .79), emergency comprehension (ω_HS_ = .37; *H* = .73), responsibility (ω_HS_ = .36; *H* = .72, knowledge (ω_HS_ = .42; *H* = .75), and intervention (ω_HS_ = .35; *H* = .70).

#### Goodness-of-fit

Robust goodness-of-fit indices with 95% CI, χ^2^/*df* ≤ 3, RMSEA ≤ 0.08, RMSR ≤ 0.08, AGFI ≥ 0.95, CFI ≥ 0.95, and NNFI/TLI ≥ 0.95 (Ferrando & Lorenzo-Seva, 2019) also indicated that a strong bifactor model fit to the data on the scale for:

- *victim offline* (RMSEA = 0.02 [95% CI: 0.00, 0.02], RMSR = 0.02 < Kelley’s value = 0.03 [0.02, 0.02], χ^2^/*df* = 114/85 = 1.34 [<3, *p* < .05], AGFI = .99 [0.99, 1.00], CFI = .99 [0.99, 1.00], and TLI/NNFI = .99 [0.99, 1.00]);
- *victim online* (RMSEA = 0.01 [95% CI: 0.11, 0.13], RMSR = 0.02 < Kelley’s value = 0.03 [0.02, 0.02], χ^2^/*df* = 102/85 = 1.20 [<3, *p* < .05], AGFI = .99 [0.99, 1.00], CFI = .99 [0.99, 1.00], and TLI/NNFI = .99 [0.99, 1.00]);
- *bystander offline* (RMSEA = 0.03 [95% CI: 0.00, 0.04], RMSR = 0.02 < Kelley’s value = 0.03 [0.02, 0.02], χ^2^/*df* = 172/85 = 2.02 [<3, *p* < .001], AGFI = .99 [0.99, 1.00], CFI = .99 [0.99, 1.00], and TLI/NNFI = .99 [0.99, 1.00]); and
- *bystander online* (RMSEA = 0.04 [95% CI: 0.02, 0.05], RMSR = 0.02 < Kelley’s value = 0.03 [0.02, 0.02], χ^2^/*df* = 242/85 = 2.84 [<3, *p* < .001], AGFI = .99 [0.99, 1.00], CFI = .99 [0.99, 1.00], and TLI/NNFI = .99 [0.99, 1.00]).

### Results for the IRT-2PL Model

The PEBI results indicated that the bifactor model was *unidimensional enough* for testing as an IRT model. As shown in Table 2, results of the IRT-2PL models indicated that discrimination parameters of the 20-item for each general factor and 4-item for each specific factor were significant with the acceptable cut-off points (α > .05, *z* > 1.96). The plots of TCC also displayed the cut-off score as 2.0 for each general and specific factor differentiating between low and high anti-bullying self-efficacy.

**Table 2.** IRT 2-PL Model and DIFDifferential Item Functioning Analyses of Outliers and Non-Outliers’ Responses to the General and Specific Factors of the Dublin Anti-Bullying Self-Efficacy Scales.

	Victim Offline				Victim Online				Bystander Offline				Bystander Online			
Factor & Item	General Factor *a*	DIF Lord’s χ^2^	Specific Factor *a*	DIF Lord’s χ^2^	General Factor *a*	DIF Lord’s χ^2^	Specific Factor *a*	DIF Lord’s χ^2^	General Factor *a*	DIF Lord’s χ^2^	Specific Factor *a*	DIF Lord’s χ^2^	General Factor *a*	DIF Lord’s χ^2^	Specific Factor *a*	DIF Lord’s χ^2^
Recognition																
Item 1	1.18*	0.06	1.59*	0.91	1.06*	0.00	1.60*	0.27	1.14*	0.31	1.60*	0.48	1.14*	0.25	1.61*	1.10
Item 2	1.14*	3.05	1.69*	0.70	1.05*	0.93	1.62*	0.20	1.12*	0.12	1.73*	0.45	1.03*	0.12	1.54*	0.14
Item 3	1.15*	0.34	1.54*	0.15	1.05*	0.32	1.51*	0.57	1.00*	1.14	1.46*	1.13	1.12*	0.43	1.56*	0.22
Item 4	1.10*	0.01	1.58*	0.16	1.02*	0.02	1.49*	2.67	1.09*	0.08	1.60*	0.00	1.02*	0.07	1.51*	0.04
Emergency Comprehension																
Item 5	1.08*	0.00	1.61*	1.67	1.06*	0.03	1.58*	1.52	1.08*	0.01	1.60*	1.06	1.02*	0.44	1.57*	1.29
Item 6	1.10*	2.34	1.80*	1.53	1.05*	0.94	1.78*	2.89	1.16*	1.69	1.74*	0.15	1.09*	0.12	1.65*	0.01
Item 7	1.08*	0.39	1.56*	0.40	1.06*	1.70	1.55*	1.21	1.09*	0.04	1.61*	1.67	1.04*	1.22	1.53*	0.43
Item 8	1.06*	0.46	1.65*	0.13	1.04*	0.19	1.78*	0.00	1.12*	0.06	1.85*	2.22	1.08*	0.27	1.52*	0.01
Responsibility																
Item 9	1.12*	0.01	1.58*	1.71	1.15*	0.01	1.58*	0.13	1.26*	0.09	2.20*	2.79	1.16*	0.33	2.11*	2.13
Item 10	1.11*	0.67	1.54*	0.06	1.15*	0.14	1.69*	0.76	1.16*	0.26	2.12*	0.01	1.13*	0.11	1.91*	2.55
Item 11	1.14*	0.10	1.63*	1.55	1.19*	0.68	1.67*	0.09	1.19*	0.33	2.17*	1.09	1.15*	0.34	2.19*	2.84
Item 12	1.16*	0.32	1.36*	0.71	1.14*	0.90	1.54*	0.31	1.17*	0.06	2.11*	1.49	1.14*	0.49	1.97*	0.02
Knowledge																
Item 13	1.12*	0.91	1.59*	0.30	1.19*	1.15	1.58*	0.41	1.14*	2.85	1.60*	0.19	1.15*	2.85	1.58*	0.27
Item 14	1.04*	3.39	1.55*	0.42	1.14*	2.62	1.59*	0.04	1.09*	1.93	1.53*	0.59	1.08*	3.35	1.54*	0.41
Item 15	1.09*	3.29	1.51*	1.35	1.22*	1.82	1.65*	1.05	1.13*	2.93	1.53*	0.08	1.09*	1.39	1.55*	0.00
Item 16	1.10*	3.15	1.63*	0.36	1.10*	3.42	1.65*	3.25	1.06*	3.04	1.64*	0.97	1.15*	2.98	1.59*	0.00
Intervention																
Item 17	1.26*	0.23	1.56*	0.13	1.29*	2.40	1.56*	1.02	1.27*	2.25	1.61*	0.03	1.29*	1.28	1.55*	0/00
Item 18	1.11*	1.21	1.47*	2.13	1.32*	1.35	1.50*	0.54	1.28*	0.63	1.61*	4.14	1.22*	2.71	1.50*	0.33
Item 19	1.26*	1.54	1.58*	1.16	1.30*	0.76	1.72*	0.01	1.27*	1.07	1.52*	0.25	1.32*	0.00	1.61*	0.13
Item 20	1.19*	0.07	1.48*	0.67	1.25*	0.70	1.65*	0.13	1.14*	0.79	1.50*	0.07	1.16*	1.54	1.50*	0.36

*Note. N* = 1,222. *Retained only items with Discrimination Parameter (α≥.5)* and without significant Nonuniform DIF (*p* < .001). DIF = Differential Item Functioning.

* *z* > 1.96.

Table 2 also shows no item had a significant DIF, indicating measurement invariance for the outlier and non-outlier groups. The results of the measurement invariance test for the gender group also showed similar non-significant DIF values but were not reported for the sake of brevity.

## Discussion

The development and measurement of anti-bullying self-efficacy beliefs are central to the prevention and/or intervention of offline and online bullying behaviors. Therefore, the effectiveness of anti-bullying programs, which is generally assessed in terms of their contributions to students’ awareness of bullying behaviors (Foody et al., 2018), also depends on the development and measurement of the anti-bullying self-efficacy beliefs of victims and bystanders (Sargioti et al., 2023). However, it was unclear the extent to which anti-bullying self-efficacy beliefs are developable and measurable as the general factor and one specific dimension. To address this issue, the current research has taken three main steps, testing the (a) unidimensional factor, (b) bifactor, and (c) IRT-2PL models. As the main result, the bifactor model fitted the data on the scale for victim offline, victim, online, bystander offline, and bystander online better than the unidimensional factor model. The bifactor model appeared to be sufficiently fitting the data on each scale with 20 items for anti-bullying self-efficacy as the general factor with five dimensions, which are *recognition, emergency comprehension, responsibility, knowledge*, and *intervention.* The research has hereby proposed a bifactor model of anti-bullying self-efficacy beliefs and sufficient evidence for the psychometric properties of the scales, with the aim of facilitating further research on the accurate evaluation of an anti-bullying program in terms of its contributions to anti-bullying self-efficacy beliefs as the general factor and one specific dimension.

Although the results, particularly ω_H_ > .80, indicated that the data could be essentially unidimensional, multidimensionality could still be considered due to five essential reasons. First, the value of ω_H_ could be high even if the data was clearly multidimensional, particularly when the number of items was large (Reise et al., 2013). Second, the values of ω_HS_ ≥ .30 (Smits et al., 2014) and *H* index ≥ .70 (Hancock & Mueller, 2001) for subscales indicated a substantial proportion of explained variance by sub-factors, which are likely to be replicable in further research. Third, a unidimensional factor solution is expected when an anti-bullying program is very effective; participants give very high scores on each scale, reducing substantial variance in item-responses. Fourth, robust goodness-of-fit indices with 95% CI indicated a strong bifactor model fit. Five, IRT-2PL model test yielded a cut-off score for low and high anti-bullying self-efficacy beliefs as the general factor and for each specific dimension, thereby allowing for measuring the extent to which the anti-bullying program was ineffective for some participants. These statistical reasons suggest that the scales can help researchers assess adolescents’ anti-bullying self-efficacy beliefs as the general factor and one specific dimension in tackling online/offline bullying behaviors as both victims and bystanders.

### Limitations

Although the present research did not address all the limitations mentioned in the earlier research (see Sargioti et al., 2023), it presented a novel way to measure the effectiveness of anti-bullying programs. It provided sufficient psychometric evidence for a bifactor model of anti-bullying self-efficacy beliefs and satisfied the criteria for construct validity and measurement invariance across groups of outlier and non-outlier as well as male and female. However, the research provided no result of measurement invariance by age, ethnicity, sexual orientation, religion and/or socioeconomic status groups, thereby not allowing for an empirical conclusion about how such diversity affects the scale development and the creation of an effective intervention program. Therefore, the research has insufficiently addressed issues in diversity, leaving it unclear whether the anti-bullying self-efficacy scales allow for comparing these groups. To address this issue warrants further testing for measurement invariance.

### Implications

The present findings have implications for a school anti-bullying policy and practice as well as for further research. The extent of victim and bystander’s self-efficacy beliefs can be used as a measure of the effectiveness of a school anti-bullying policy and program across countries, including Ireland (Sargioti et al., 2023). To measure the extent to which an anti-bullying program is ineffective for some participants or whether outliers are statistically representative of the student population attending that program, the cut-off score of 2.0 can be used to differentiate between low and high anti-bullying self-efficacy beliefs. Below-average victims and bystanders are expected to give a maximum score of 2.0 for each general and specific factor, whereas those above-average would have a mean score of 2.1 and above.

The proposed bifactor model allows further research to assess adolescents’ anti-bullying self-efficacy beliefs in tackling both offline and online bullying situations as the general factor and specific sub-factor. Further research can operationalize the anti-bullying self-efficacy concept as the general factor alone, or focus on one sub-dimension only (recognition, emergency comprehension, responsibility, knowledge, and intervention), or both at the same time. The research hereby provides theoretical and empirical steps forward in the anti-bullying literature as it allows measuring weaknesses and strengths of both specific and general anti-bullying self-efficacy beliefs.

One essential recommendation for further research concerns using outliers as one criterion variable for distinguishing between participants for whom the anti-bullying program was effective and ineffective. Outliers are very likely to be participants who give the lowest or very low scores on each scale, indicating the program ineffectiveness for them. Outliers can inflate an inter-factor correlation value, as it happened in the current research. Therefore, if they are not removed, further factor analysis might not yield the five-factor solution. However, given that outliers are peculiar to a dataset for factor analysis, a standardized score for outlier detection/removal may facilitate the replicability of the five-factor model. One standardized method is to use *regression factor scores* as they show the location of each participant’s relative standing on a latent factor (DiStefano et al., 2009). To use a specific range of regression factor scores in comparison with Boxplot results could allow further research to test replicability of the five-factor. The present research detected a regression factor score ≥ −2.0 (i.e., two SDs below the mean) as the cut-off point inflating inter-factor correlations in the current dataset, and compared it with Boxplot results, thereby excluded outliers on both tests. This cut-off score could be considered or tested in further research.

## Conclusions

The current research made novel theoretical and empirical contributions to the anti-bullying literature by allowing to measure the weaknesses and strengths of both specific and general anti-bullying self-efficacy beliefs that might occur as a result of the entire anti-bullying program. The research proposed a bifactor model of anti-bullying self-efficacy beliefs and provided statistical evidence for the psychometric properties of the four scales measuring the bifactor structure. As the main implications for further research, victim and bystander’s anti-bullying self-efficacy could be operationalized/measured as either the general or specific factor alone (i.e., recognition, emergency comprehension, responsibility, knowledge, and intervention), or both at the same time.
